# Comparison of robotic-assisted and laparoscopic-assisted natural orifice specimen extraction surgery in short-terms outcomes of middle rectal cancer

**DOI:** 10.1186/s12957-023-03083-w

**Published:** 2023-07-04

**Authors:** Shan-ping Ye, Hong-xin Yu, Dong-ning Liu, Wei-jie Lu, Can Wu, Hao-cheng Xu, Tai-yuan Li

**Affiliations:** Department of General Surgery, The First Affiliated Hospital of Nanchang University, No. 17 Yongwaizheng Street, Nanchang, 330006 Jiangxi Province China

**Keywords:** Robotic NOSES, Laparoscopic NOSES, Middle rectal cancer, Short-time outcomes, Anal function

## Abstract

**Background:**

Surgery is becoming less invasive as technology advances. Natural orifice specimen extraction surgery (NOSES) ushered in a new era of minimally invasive techniques. At the same time, NOSES is gaining popularity in the world. With their distinct advantages, surgical robots have advanced the development of NOSES. The aim of current study was to compare the short-term outcomes between robotic-assisted NOSES and laparoscopic-assisted NOSES for the treatment of middle rectal cancer.

**Methods:**

Patients with middle rectal cancer who underwent robotic-assisted or laparoscopic-assisted NOSES at the First Affiliated Hospital of Nanchang University between January 2020 and June 2022 had their clinicopathological data collected retrospectively. 46 patients were enrolled in the study: 23 in the robotic group and 23 in the laparoscopic group. Short-term outcomes and postoperative anal function in the two groups were compared.

**Results:**

There was no significant difference in the clinicopathological data between the two groups. The robotic group had less intraoperative blood loss (*p* = 0.04), less postoperative abdominal drainage (*p* = 0.02), lower postoperative white blood cell counts (*p* = 0.024) and C-reactive protein levels (*p* = 0.017), and shorter catheter removal time when compared to the laparoscopic group (*p* = 0.003). Furthermore, there were no significant difference in mean operative time (159 ± 31 min vs 172 ± 41 min) between the robotic and laparoscopic groups (*p* = 0.235), but time to naked the rectum (86.4 ± 20.9 min vs. 103.8 ± 31.5 min *p* = 0.033) and time of digestive tract reconstruction (15.6 ± 3.88 min vs. 22.1 ± 2.81 min *p* < 0.01) in the robotic group were significantly shorter than laparoscopic group. The robotic group had lower postoperative Wexner scores than the laparoscopic group.

**Conclusions:**

This research reveals that combining a robotic surgical system and NOSES results in superior outcomes, with short-term outcomes preferable to laparoscopic-assisted NOSES.

## Background

Colorectal cancer, the third most common cancer worldwide, is also associated with a very high mortality rate [1, 2] and poses a significant challenge to public health. Surgery remains one of the most important methods for colorectal cancer treatment. Numerous studies have confirmed the safety and feasibility of laparoscopic surgery as a minimally invasive technique for colorectum treatment [3, 4], but it is not without limitation [5, 6]. In recent years, the popularity of robotic colorectal cancer surgery has been rising. With high-definition lens and flexible robotic arm, the robotic surgery system can greatly remove the tremor of the operator’s hand, improve the flexibility and accuracy of the operator’s operation, and robotic surgery is more conducive to difficult operations in confined spaces. In some aspects, such as postoperative patient voiding function, sexual function, and surgical complications, robotic surgical systems have been shown to be superior to laparoscopy in some reports [7–9].

NOSES, as an emerging minimally invasive technique, has got the interest of the minimally invasive surgery community, particularly in colorectal surgery [10, 11]. Compared to transabdominal specimen extraction surgery, NOSES improves patients’ psychological health [12], at the same time NOSES has favorable short-term outcome [13–15]. NOSES can rely on both robotic and laparoscopic methods. In recent years, there have been an increasing number of studies available on the comparison of NOSES with conventional surgery. However, researches on robotic-assisted NOSES versus laparoscopic-assisted NOSES are rare. Therefore, we conducted this study to compare the short-term outcomes of the two surgical approaches.

## Methods

### Study population and data collection

In this retrospective cohort study, we retrospectively collected and analyzed clinicopathological data from patients who underwent middle rectal cancer surgery with NOSES at the First Affiliated Hospital of Nanchang University between January 2020 and June 2022. Referring to the Chinese Protocol of Diagnosis and Treatment of Colorectal Cancer (2020 edition), When the distance from the lower edge of the tumor to the anal margin is 5–10 cm, it is considered a middle rectal cancer. NOSES was performed on 50 patients with middle rectal cancer, there were 26 patients in the robotic NOSES group and 24 patients in the laparoscopic NOSES group. Four patients were excluded due to combined liver metastases, ASA IV, preoperative chemotherapy and combined splenectomy, while 46 patients met the criteria, with 23 in the robotic group and 23 in the laparoscopic group. The research was approved by the ethics committee of the First Affiliated Hospital of Nanchang University and complied with the relevant requirements in the Declaration of Helsinki.

Inclusion criteria: (1) age greater than 18 years and less than 80 years. (2) pathologically confirmed primary rectal adenocarcinoma on endoscopic biopsy. (3) signed informed consent. (4) Confirmation of tumor location in the middle rectum based on the imaging, colonoscopy, intraoperative findings, and postoperative pathology.

Exclusion criteria: (1) concurrent other malignancies or distant metastasis. (2) Cases with emergency surgery due to bleeding, obstruction, or perforation. (3) Transit open surgery. (4) Incomplete data or missing follow-up data. (5) Combined organ resection. (6) Preoperative chemoradiotherapy. (7) American Society of Anesthesiologists (ASA) classification > III.

### Surgical technique

The patients’ position and trocar position can be referred to our previous study [16]. Robotic NOSES has the same procedure as laparoscopic NOSES. After the rectum and its mesorectum were dissociated, the rectum was transected at 2 cm below the tumor by using a linear stapler. Then the rectal stump was incised and disinfected with iodophor, the protective sleeve was placed into the abdominal cavity through the assistant hole. An assistant delivered oval forceps into the pelvic cavity through the anus and used oval forceps to grip one end of the protective sleeve. Then slowly pulled out the protective sleeve. Eventually, one end of the protective sleeve was placed inside the abdominal cavity and the other outside the anus, completely covering the rectal stump and the perianal area. Tumor was pulled out of the rectal stump, then the colon was then disconnected at 10 cm above the tumor. The anvil was placed into the stump of the sigmoid colon and disinfected with iodophor, and then the anvil was delivered into the abdominal cavity. The rectal stump was sutured with purse-string suture. Circular stapler was placed transanally, end-to-end anastomosis of the rectum and sigmoid colon is performed. After completion of digestive tract reconstruction. The pelvic and abdominal cavities were washed repeatedly with normal saline until there were no blood remained. Then, all lavage fluid was removed. Finally, using normal saline (500 ml) to wash the pelvic and abdominal cavities again, the lavage fluid was aspirated into sterile bottle.

### Parameters of observation and evaluation

The general demographic data of patients included age, gender, body mass index (BMI, kg/m^2^), and American Society of Anesthesiologists (ASA) classification. The pathological data of patients include distance of tumor and anal, diameter of neoplasm, number of harvested lymph nodes, perineural invasion, lymphatic or vascular invasion, Positive margin and TNM stage (using the 8th edition of the AJCC TNM staging system for colorectal cancer). The surgical parameters for the patients were as follows: total operative time, time to naked the rectum (defined as the time from the completion of surgical instrument installation to the complete nakedness of the rectum and its mesentery), time of specimen removal (defined as the time from the complete nakedness of the rectum to the removal of the specimen), time of digestive tract reconstruction (defined as the time from the removal of the specimen to the completion of anastomosis) and intraoperative blood loss. White blood cell counts and C-reactive protein levels were applied to assess postoperative inflammatory responses. Using 10 ml of lavage fluid in the sterile bottle for bacterial culture and the remaining lavage fluid for ascitic cancer cell examination. The Clavien-Dindo classification was used to record postoperative complications. The Wexner score evaluates patients’ anal function three months after surgery.

### Statistical analysis

All statistical analyses were performed using SPSS 22.0. All parameters were tested for normality, with data from a normal distribution expressed as mean ± SD and non-normal data expressed as median and range, respectively, using the independent samples t-test or Mann–Whitney *U* test. Categorical data were expressed as frequencies and percentages, and the *χ* ^2^ test or Fisher’s exact probability method were used to calculate them. *P* < 0.05 was regarded as statistically significant.

## Results

### Clinical baseline characteristics

The gender, age, BMI, preoperative white blood cell counts, levels of preoperative C-reactive protein, distance between tumor and anal, diameter of neoplasm, TNM stage, and ASA classification of the patients were compared in this study, and no significant differences in clinical baseline characteristics between the two groups (*p* > 0.05) (Table 1).

**Table 1:** Comparison of baseline data between robotic group and laparoscopic group

	Robot (*n* = 23)	Laparoscope (*n* = 23)	*p*
Gender (*n*, %)			0.78
Male	11(47.8%)	12(52.2%)	
Female	12(52.2%)	11(47.8%)	
Age (mean ± SD, years)	57 ± 10.39	61 ± 8.58	0.206
BMI (mean ± SD, kg/m^2^)	22.00 ± 2.56	23.26 ± 2.48	0.098
CRP (mean ± SD, mg/L)	5.51 ± 5.3	4.06 ± 2.86	0.254
WBC (mean ± SD, /L)	5.45 ± 1.39	5.68 ± 1.62	0.607
Distance of tumor and anal (mean ± SD, cm)	7.8 ± 1.88	7.4 ± 1.16	0.439
Diameter of neoplasm (mean ± SD, cm)	3.1 ± 1.05	2.8 ± 1.20	0.402
TNM stage (*n*, %)			0.624
I	8(34.8%)	9(39.1%)	
II	9(39.1%)	6(26.1%)	
III	6(26.1%)	8(34.8%)	
ASA (*n*, %)			0.326
II	8(34.8%)	5(21.7%)	
III	15(65.2%)	18(78.3%)	

*BMI* body mass index, *CRP* C-reactive protein, *WBC* white blood cell

### Comparison of perioperative indexes between robotic group and laparoscopic group

**Fig. 1:**
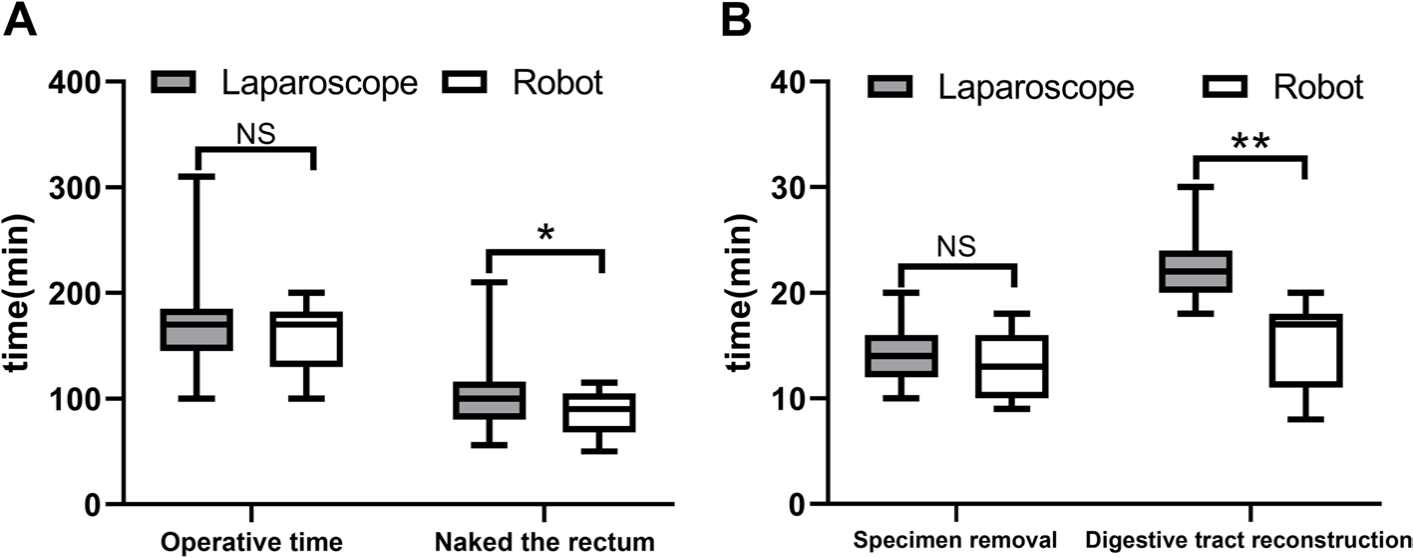
To compare the operative time in the robotic and laparoscopic groups. **A** Total operative time (*p* > 0.05) and time to naked the rectum (*p* < 0.05). **B** Time of specimen removal (*p* > 0.05) and time of digestive tract reconstruction (*p* < 0.01). NS:*p* > 0.05, *:*p* < 0.05, **:*p* < 0.01

**Fig. 2:**
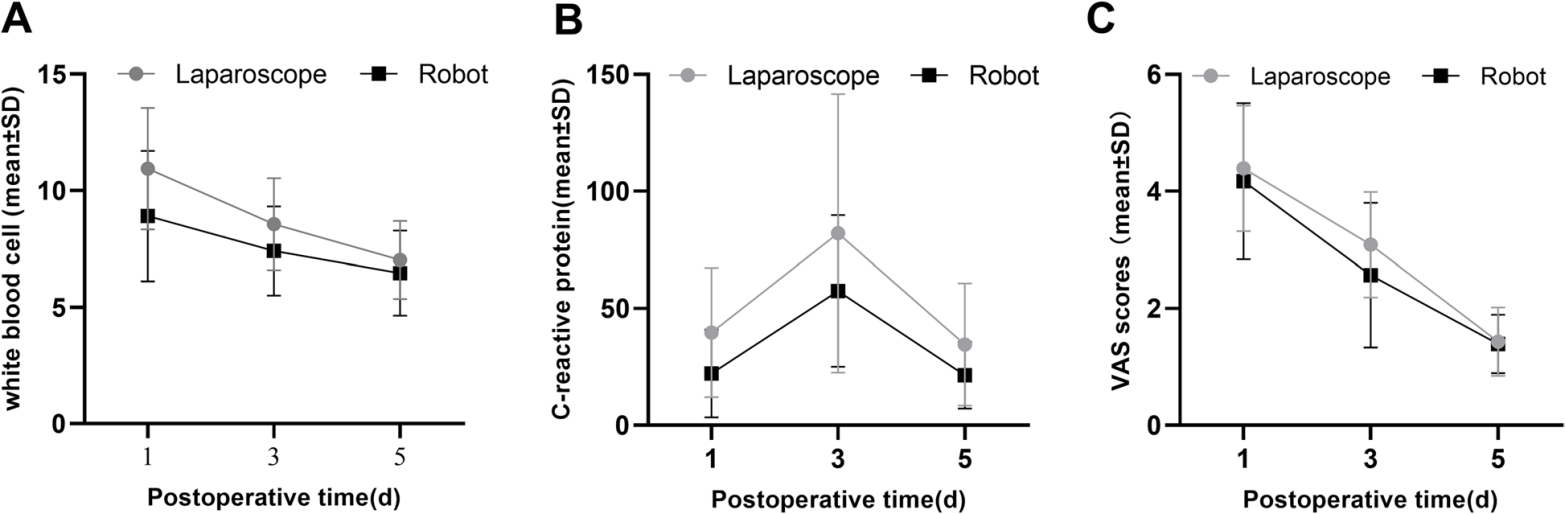
Comparison of perioperative indexes between two groups. **A** White blood cells cores (*p* = 0.017). **B** C-reactive protein scores (*p* = 0.024). **C** VAS scores (*p* = 0.309)

Table 2 demonstrates a comparison of perioperative data between the robotic and laparoscopic group. The operative time of 159 ± 31 min in robotic group was similar to that of 172 ± 41 min in laparoscopic group (*p* = 0.235), However, compared to the laparoscopic group, the time to naked the rectum (robotic group 86.4 ± 20.9 min vs. Laparoscopic group 103.8 ± 31.5 min mp = 0.033) and the time of digestive tract reconstruction (robotic group 15.6 ± 3.88 min vs. laparoscopic group 22.1 ± 2.81 min *p* < 0.01) (Table 2, Fig. 1) were shorter in robotic group. And there had less intraoperative bleeding in the robotic group (robotic group 79.5 ± 32.3 ml vs. laparoscopic group 106 ± 25.9 ml, *p* = 0.04). In indicators of post-operative recovery, time to first flatus (robotic group 60 ± 7 h vs. laparoscopic group 64 ± 8 h *p* = 0.09) and time to liquid diet (robotic group 72.1 ± 6.5 h vs. laparoscopic group 75.1 ± 7.16 h *p* = 0.157) were similar in both groups. Postoperative VAS scores (Table 3, Fig. 2C) and the postoperative hospital stay (robotic group 10.9 ± 5.5 days vs. laparoscopic group 10.7 ± 5.4 days *p* = 0.936) were similar in two groups, The postoperative volume of abdominal drainage was less in the robotic group (robotic group 276 ± 63 ml vs. laparoscopic group 327 ± 77 ml *p* = 0.02). Comparison of postoperative inflammation-related indicators. Postoperative white blood cell counts and levels of C-reactive protein were lower in the robotic group than in the laparoscopic group (*p* = 0.024; *p* = 0.017) (Table 3, Fig. 2A, B). Regarding postoperative complications, two complications occurred in the robotic group and four in the laparoscopic group.

**Table 2:** Comparison of perioperative indexes between robotic group and laparoscopic group

	Robot (*n* = 23)	Laparoscope (*n* = 23)	*p*
Total operative time, mean (SD), min	159(31)	172(41)	0.235
Time to naked the rectum, mean (SD), min	86.4(20.9)	103.8(31.5)	0.033
Time of specimen removal, mean (SD), min	13.2(2.69)	14.4(2.59)	0.125
Time of digestive tract reconstruction, mean (SD), min	15.6(3.88)	22.1(2.81)	< 0.01
Estimated blood loss, mean (SD), ml	79.5(32.3)	106(25.9)	0.04
Time to first flatus, mean (SD), h	60(7)	64(8)	0.09
Time to liquid diet, mean (SD), h	72.1(6.50)	75.1(7.16)	0.175
Time to remove urinary catheter, mean (SD), day	3.67(0.71)	4.43(0.95)	0.003
Postoperative volume of abdominal drainage, mean (SD), ml	276(63)	327(77)	0.02
Postoperative hospital stays, mean (SD), day	10.9(5.5)	10.7(5.4)	0.936
Bacteriological examination of the ascites, *n*	0	0	NA
Ascitic cancer cell examination, *n*	0	0	NA
Harvested lymph nodes, *n* (%)			0.305
< 12	6(24)	9(37.5)	
≥ 12	19(76)	15(62.5)	
Perineural invasion, *n* (%)	5(21.7)	3(13)	0.646
Lymphatic or vascular invasion, *n* (%)	5(21.7)	5(21.7)	1
Positive margin, *n*	0	0	NA
Postoperative complication, *n* (%)	2(8.7)	4(17.3)	0.062
Pneumonia	1	1	
Intestinal obstruction	0	1	
Anastomotic leakage	1	1	
Intra-abdominal infection	0	1	
Complication of Clavien-Dindo classification ≥ 3, *n*	0	0

**Table 3:** Comparison of postoperative C-reactive protein, white blood cell and VAS scores between robotic group and laparoscopic group

	Robot (*n* = 23)	Laparoscope (*n* = 23)	*p*
Postoperative white blood cell, mean (SD), count /L			0.024
Day 1	8.90(2.79)	10.93(2.61)	
Day 3	7.40(1.90)	8.55(1.97)	
Day 5	6.45(1.82)	7.01(1.67)	
Postoperative C-reactive protein, mean (SD), mg/L			0.017
Day 1	22.18(18.73)	39.67(27.66)	
Day 3	51.48(32.46)	82.07(59.57)	
Day 5	21.38(14.30)	34.54(26.15)	
VAS scores, mean (SD)			0.309
Day 1	4.17(1.33)	4.39(1.07)	
Day 3	2.56(1.24)	3.08(0.90)	
Day 5	1.39(0.50)	1.43(0.59)	

*Day 1* first day after surgery, *Day 3* third day after surgery, *Day 5* fifth day after surgery

The* P* value was calculated by repeated measures statistical analysis

### Comparison of postoperative anal function in the robotic and laparoscopic groups

At 3 months after surgery, the robotic group had a lower Wexner score than the laparoscopic group, and the robotic group had better anal function (Table 4, *p* = 0.024).

**Table 4:** Comparison of Wexner scores between robotic group and laparoscopic group

Type of incontinence	Robot (*n* = 23)	Laparoscope (*n* = 23)	*p*
Solid	2(1–3)	2(1–3)	
Liquid	2(1–3)	2(1–3)	
Gas	2(1–3)	2(1–4)	
Wears pad	1(0–2)	2(1–2)	
Lifestyle alteration	2(1–3)	2(1–3)	
Total score	9(5–13)	11(7–14)	0.001

The *P* value was calculated by Mann–Whitney *U* test

## Discussion

Conventional laparoscopic proctocolectomy requires an auxiliary abdominal incision to remove the specimen, at the same time facing a series of problems associated with the abdominal incision, such as incisional infection, postoperative pain, incisional hernia, and the psychological impact of abdominal scarring. NOSES can effectively avoid these problems. There are only several small scars after NOSES, smaller surgical incisions may mean lower rates of incision complication [17]. Laparoscopic-assisted NOSE is extremely difficult and demanding for the operators. For difficult operations in confined spaces, such as the pelvis, robotic surgical systems are preferable. Combining the benefits of robotic surgical systems and NOSES may result in a better outcome. Because there have few studies comparing robotic-assisted versus laparoscopic-assisted NOSES for middle rectal cancer, we conducted this study to compare the short-term outcomes of two surgeries. Our studies revealed that robot-assisted NOSES significantly reduced the time to naked the rectum as well as the time required for digestive tract reconstruction, meanwhile robotic-assisted NOSES had less intraoperative blood loss, lower postoperative CRP and WBC levels, shorter time to ureter removal. Furthermore, robotic-assisted NOSES has better anal function after surgery.

The total operative time was similar in both groups (*p* = 0.235), The same results were reported in a meta-analysis by Trastulli et al. [18], but the robotic group showed a significant reduction in time to naked rectum (*p* = 0.033) and time of digestive tract reconstruction (*p* < 0.01). The total operative time was similar in both groups, which could be attributed to the more complex setup and docking of the robotic surgical system, which took a relatively long time. At the same time, the robotic surgical system’s highly flexible robotic arm and clearer field of view are more conducive to complex and delicate operations, which may account for the remarkably shorter time to naked rectum and time of digestive tract reconstruction. These advantages of robots were also recognized in several studies [19, 20].

One of the most important indicators of surgical quality is intraoperative bleeding. In our research, intraoperative bleeding (*p* = 0.04) was significantly less in robotic-assisted NOSES. This is similar to the results of a multicenter randomized controlled study in which our center participated [21], and the amount of intraoperative bleeding was related to surgical area vascular protection. In this regard, robotic surgical systems are more advantageous [22]. Postoperative abdominal drainage was mainly generated by exudate from the surgical area. The robotic surgical system can reduce the damage to body tissues, less surrounding fat and other tissue residues; therefore, the postoperative abdominal drainage were less in the robotic group.

Postoperative inflammatory response is important indicators of surgical quality and postoperative recovery [23]. We used white blood cells and plasma C-reactive protein levels to assess the postoperative inflammatory response. White blood cells (*p* = 0.024) and plasma C-reactive protein levels (*p* = 0.017) were significantly lower in the robotic group in our study. This were most likely due to the robot’s shorter intra-abdominal operational time and the organism's lower stress response. According to some studies, inflammation is one of the factors that promote tumorigenesis and metastasis [24, 25], and a lower inflammatory response following robotic-assisted NOSES may be better for patient prognosis.

In terms of surgical safety, there was no significant difference in the incidence of postoperative complications between the two groups (*p* = 0.062). Leroy et al. [26] reported 16 patients with sigmoid diverticulitis who underwent NOSES, and bacterial cultures of ascites were positive in all patients. In the present study, after the completion of the digestive tract reconstruction, We flushed the abdominal cavity with a large amount of saline. In either group, no positive results were found in the bacterial culture of postoperative ascites. Furthermore, no cancer cells were detected in the postoperative abdominal drainage fluid. The similar finding was reported in research by Ngu et al. [27]. In terms of tumor radicalization, the two groups had similar numbers of harvested lymph nodes (*p* = 0.305) and positive cut margins.

On the question of whether NOSES can cause anal impairment. With the continuous standardization of the NOSE [28], it does not affect anal function when the indications for the NOSES are strictly followed [29]. And in our research, the postoperative anal function was greater in the robotic group compared to the laparoscopic group, The use of robotic surgical systems may better protect anal function which was also reported in the meta-analysis by Grass et al. [30]. In the meta-analysis, Broholm et al. [31] reported that patients had a better urological function after robotic surgery. Similar results emerged in our study, there had a shorter Indwelling urinary catheter time after robotic-assisted NOSES. Grass et al. [30] and Broholm et al. [31] thought that robotic surgical system has optimized visualization and flexible instruments with multiple degrees of freedom which facilitate the identification and preservation of nerves [32].

Finally, our study has some limitations. Because this is a retrospective study, selection bias is unavoidable, and our sample size is small due to the single-center study and the limitations of the surgical approach. More randomized controlled studies with a larger sample size are thus required for further investigation.

## Conclusions

In summary, robotic-assisted NOSES is a safe and feasible minimally invasive technique. In comparison to laparoscopic-assisted NOSES, robotic-assisted NOSES can achieve similar radical results, while robotic-assisted NOSES had better short-term outcomes, including less operative blood loss, reduced time of intra-abdominal operation and postoperative inflammatory reaction, less abdominal drainage, shorter time to ureter removal, better intraoperative vessel and nerve preservation, better postoperative anal function and quality of life. Patients with middle rectal cancer benefit more from the combination of robotic surgical system and NOSES.

## Data Availability

Access to the database can be obtained from the corresponding author on reasonable request.

## Abbreviations

ASA: American Society of Anesthesiologists
BMI: Body Mass Index
CRP: C-reactive protein
NOSES: Natural orifice specimen extraction surgery
VAS: Visual analog scale
WBC: White blood cell
